# Peak atrial longitudinal strain is predictive of atrial fibrillation in patients with chronic obstructive pulmonary disease and coronary artery disease

**DOI:** 10.1111/echo.15074

**Published:** 2021-05-10

**Authors:** Rita Pavasini, Gioele Fabbri, Alessio Fiorio, Roberta Campana, Giulia Passarini, Filippo Maria Verardi, Marco Contoli, Gianluca Campo

**Affiliations:** ^1^ UO Cardiologia Azienda Ospedaliero Universitaria di Ferrara Ferrara Italy; ^2^ Department of Translational Medicine University of Ferrara Ferrara Italy; ^3^ GVM Care & Research Maria Cecilia Hospital Cotignola Italy

**Keywords:** atrial fibrillation, chronic obstructive pulmonary disease, left atrial longitudinal strain, peak atrial longitudinal strain, stroke

## Abstract

**Background:**

The peak atrial longitudinal strain (PALS) has been validated in the prediction of atrial fibrillation (AF) in the general population. If this finding can be applied to patients with chronic obstructive pulmonary disease (COPD) and concomitant coronary artery disease (CAD) is unknown.

**Methods and results:**

We analyzed two different study populations of patients with COPD and acute CAD in SCAP trial (Clinical trial.org identifier NCT02324660) and COPD and stable CAD in the NATHAN‐NEVER trial (clinical trial.org identifier NCT02519608). All patients enrolled underwent spirometry and clinical specialistic evaluation to test COPD diagnosis. During the index evaluation, all patients underwent echocardiography. The primary endpoint of the study was the occurrence of AF. Overall, 175 patients have been enrolled. PALS was significantly lower in patients with COPD compared to patients without COPD (26% ± 8% vs. 30% ± 8% for PALS4CV, *P* = .003). After a mean follow‐up of 49 ± 15 months, 26 patients experienced at least one episode of AF. At multivariable analysis, only PALS (HR: 0.92, 95% CI: 0.86‐0.98, *P* = .014) resulted as an independent predictor of AF in COPD patients with CAD, with the best cutoff value of 25.5% (sensitivity 87% and specificity 70%).

**Conclusion:**

The present study confirmed a high incidence of AF events in COPD patients and that PALS is altered and able to independently predict AF in a specific cohort of patients with CAD and COPD. This study points out the need to integrate PALS measurement in the echocardiographic workup of all COPD patients, to early identify those at high risk of AF development.

## 1 INTRODUCTION

Cardiovascular diseases (CVDs) are the most prevalent comorbidities in chronic obstructive pulmonary disease (COPD). Not only are CVDs frequent in COPD patients, but they also contribute to disease severity and poor prognosis.^1^, ^2^ Coronary artery disease (CAD) and atrial fibrillation (AF) represent the most prevalent CVDs in COPD patients^1^ being mutually related diseases as CAD promotes the development and progression of AF.^2^ In particular, patients with COPD have a 28% increased risk of developing AF, especially during exacerbations.^3^ At the same time, the Atherosclerosis Risk in Communities (ARIC) study showed that the rate of incident AF is inversely associated with flow expiratory volume at first second (FEV1).^4^ Previously, by means of specific echocardiographic parameters able to explore right heart function (namely fractional area change and right ventricle strain), we found that smoking patients admitted to hospital for acute coronary syndromes (ACS) with concomitant and previously undiagnosed COPD showed higher right heart dysfunction compared to patients with ACS but without COPD.^5^ The data would suggest that COPD can affect right heart functionality through different mechanisms, including pulmonary hypertension and lung hyperinflation, mainly because of hypoxia. At the same time, hypoxia might justify the higher prevalence of atrial fibrillation in patients with COPD that usually have also left atrium dilatation and increased left ventricle (LV) filling pressure that are not consequence of systolic dysfunction, rather to hypoxia‐related tachycardia.^6^ Secondly, an animal study showed that rabbit with COPD had higher left atrium fibrosis, justifying the increase in atrial fibrillation.^7^ The peak atrial longitudinal strain (PALS) during the reservoir phase is a new and adjunctive parameter of the atrial function that reflects the amount of atrial fibrosis and stiffness, factors implicated in atrial fibrillation susceptibility.^8^ Previous studies showed that PALS is superior to conventional echocardiographic atrial parameters in predicting AF in the general population.^8^ If this finding can be applied to COPD patients with concomitant CAD is unknown. If confirmed, PALS could help discriminate COPD patients at higher risk of developing AF and could contribute to an early diagnosis before embolic complications. Therefore, the aim of the present analysis was to investigate the role of PALS in patients with COPD and CAD in the prediction of AF.

## 2 METHODS

### 2.1 Study population

The present analysis was carried out by using two different study populations: Screening for COPD in ACS Patients (SCAP) trial (clinical trial.org identifier NCT02324660) and The comparisoN between ticAgrelor and clopidogrel effect on endoTHelial, platelet ANd iNflammation parameters in patiEnts with stable coronary artery disease and chronic obstructiVE pulmonaRy disease undergoing percutaneous coronary intervention (NATHAN‐NEVER) trial (clinical trial.org identifier NCT02519608).^9^, ^10^ The SCAP trial was a prospective, single‐center, investigator‐driven study enrolling 137 consecutive patients admitted to hospital for acute coronary syndrome (ACS) with a smoking habit history (smoker or former smoker) between December 2014 and August 2015. Two months after discharge, all patients underwent spirometry and clinical specialistic evaluation to determine the presence of COPD.^9^ In the NATHAN‐NEVER trial, 58 patients were enrolled, with stable CAD with an established diagnosis of stable COPD (confirmed by spirometry and clinical specialistic evaluation) between September 2015 and June 2016.^10^ Both studies (SCAP and NATHAN‐NEVER) were approved by the local ethical review board and were conducted in accordance with the ethical principles of the Declaration of Helsinki. Patients were informed that their participation was voluntary, and all gave informed written consent.^9^, ^10^

### 2.2 Echocardiography

Patients enrolled in the two studies underwent a comprehensive transthoracic (TTE) echocardiography during the index hospitalization. TTEs have been performed with GE Vivid 7 and E9 (GE Healthcare) with 3.5 MHz or M5S transducers. The following parameters have been calculated according to current guidelines^11^: Ejection fraction (EF) calculated by the modified biplane Simpson method, left atrial volume indexed for body surface area (BSA), end‐diastolic and end‐systolic LV volume (EDV and ESV), diastolic dysfunction degree according to the 2016 American Society of Echocardiography (ASE)/ European Association of Cardiovascular Imaging proposed algorithm,^12^ right ventricle (RV) fractional area change (FAC), and tricuspid annular plane systolic excursion (TAPSE). Two‐dimensional speckle tracking analysis was performed off line. Global longitudinal strain (GLS) of the LV calculated from the apical views; strain of the free wall of the RV (RVS) was calculated by the apical four‐chamber view (4CV). PALS was calculated in four‐ and two‐chamber view (4 and 2CV), and mean values (±standard deviation) were obtained. During apnea, two cardiac cycles have been recorded for every image and then stored in cine‐loop format with a frame rate between 50 and 70 Hz. The postprocessing analyses have been performed with EchoPAC software version 202 (GE Healthcare). The intra‐ and inter‐rater agreement between operators was tested with Bland–Altman scatter plot analysis and intra‐class correlation coefficients on the first 20 TTEs analyzed and showing good reproducibility.

### 2.3 Clinical follow‐up

All patients underwent periodic clinical visits, and the last follow‐up contact was in November 2020 (mean follow‐up 49 ± 15 months). The primary endpoint of the present study was the occurrence of at least one episode of AF in patients with CAD and concomitant COPD. The occurrence of stroke was also assessed.

### 2.4 Statistical analysis

Continuous data were tested for normal distribution with the Kolmogorov–Smirnov test. Normally distributed values were presented as mean ± SD and compared by *t* test. Otherwise, median [interquartile range] and Mann–Whitney U were used. Categorical variables were summarized in terms of counts and percentages and were compared by using the two‐sided Fisher's exact test.

Univariate and multivariable Cox regression analysis was performed to assess the prognostic value of PALS for AF in COPD patients. Clinical and echocardiographic variables included in Table 1 with *P* < .1 after stratification for the presence of atrial fibrillation in patients with CAD and COPD were included in univariate analysis and those variables with *P* < .05 at univariate analysis were included in multivariable analysis. For Cox regression analysis, PALS calculated in 4CV was used (PALS4CV). The area under the receiver operating characteristic (ROC) curve (AUC) and Youden index was also analyzed to find the best cutoff value of PALS to predict AF in COPD patients. All statistical analyses were performed with Stata Stata/SE version 16 software (Stata Corp).

**TABLE 1:** Clinical and echocardiographic characteristics of the study population

	Total (n = 175)	COPD			COPD patients stratified according to the occurrence of AF during the follow‐up		
		No (n = 85)	Yes (n = 90)	*P*	No (n = 67)	Yes (n = 23)	*P*
Age at baseline, (y)	67.25 ± 9.91	64.74 ± 9.73	69.62 ± 9.54	.**001**	68.35 ± 9.81	73.31 ± 7.76	.**030**
Male sex, no. (%)	148 (85)	70 (82)	78 (87)	.43	58 (87)	20 (87)	.96
BMI, (Kg/m^2^)	27.21 ± 4.43	27.67 ± 4.28	26.77 ± 4.54	.18	26.19 ± 4.70	28.44 ± 3.64	.**040**
Current smoker, no. (%)	79 (45)	41 (48)	38 (42)	.42	32 (48)	6 (26)	.069
Previous smoker, no. (%)	93 (53)	44 (52)	49 (54)	.72	33 (49)	16 (70)	.091
Pack/years	39.17 ± 29.61	33.55 ± 22.60	44.47 ± 34.25	.**014**	42.53 ± 31.58	50.13 ± 41.34	.36
Hypertension, no. (%)	122 (70)	57 (67)	65 (72)	.46	49 (73)	16 (70)	.74
Hyperlipidemia, no. (%)	98 (56)	47 (55)	51 (57)	.85	40 (60)	11 (48)	.32
Diabetes, no. (%)	42 (24)	16 (19)	26 (29)	.12	19 (28)	7 (30)	.85
Acute coronary syndrome, no. (%)	134 (77)	85 (100)	49 (54)	**<.001**	34 (51)	15 (65)	.23
AF during follow‐up, no. (%)	27 (15)	4 (5)	23 (26)	**<.001**	‐‐	‐‐	‐‐
Angiographic data							
Multivessel PCI	61 (35)	32 (38)	29 (32)	.45	20 (30)	9 (39)	.41
Target vessel of PCI							
LM	21 (12)	12 (14)	9 (10)	.40	5 (7)	4 (17)	.17
LAD	91 (52)	42 (49)	49 (54)	.51	37 (55)	12 (52)	.80
LCx	60 (34)	34 (40)	26 (29)	.12	18 (27)	8 (35)	.47
RCA	63 (36)	40 (47)	23 (26)	.06	15 (22)	8 (35)	.24
Cardiovascular therapy, no. (%)							
Aspirin	174 (99)	84 (99)	90 (100)	.99	67 (100)	23 (100)	.99
P2Y12 inhibitors	175 (100)	85 (100)	90 (100)	.99	67 (100)	23 (100)	.99
Beta blocker	147 (84)	72 (85)	75 (83)	.80	56 (84)	19 (83)	.91
ACE inhibitor/ARB antagonist	162 (93)	77 (91)	85 (94)	.33	65 (97)	20 (87)	.069
Statin	170 (97)	83 (98)	87 (97)	.70	64 (96)	23 (100)	.30
Left heart							
EDV, mL	115.73 ± 43.76	112.42 ± 35.39	118.86 ± 50.41	.33	114.27 ± 52.28	132.22 ± 42.81	.14
ESV, mL	54.61 ± 35.68	51.73 ± 27.23	57.32 ± 42.11	.30	53.98 ± 44.64	67.06 ± 32.61	.20
EF, %	54.88 ± 10.24	55.41 ± 9.98	54.37 ± 10.50	.50	55.55 ± 9.84	50.95 ± 11.78	.069
LV GLS, %	−15.06 ± −4.35	−14.35 ± −4.06	−15.81 ± −4.54	.**031**	−16.73 ± −4.21	−13.04 ± −4.47	.**001**
LAVol i, mL/m^2^	31.46 ± 15.71	28.05 ± 6.48	34.62 ± 20.46	.**007**	34.78 ± 22.75	34.16 ± 11.54	.91
Diastolic dysfunction				.26			.67
Degree 0–1 (%)	115 (66)	61 (72)	54 (60)		42 (63)	12 (52)	
Degree 2–3 (%)	22 (13)	9 (11)	13 (14)		9 (13)	4 (17)	
Undetermined	38 (22)	15 (18)	23 (26)		16 (24)	7 (30)	
Avg PALS	27.53 ± 7.83	29.31 ± 7.67	25.85 ± 7.64	.**003**	28.11 ± 6.56	19.29 ± 6.81	**<.001**
4CV PALS, %	28.05 ± 8.25	29.92 ± 8.08	26.28 ± 8.06	.**003**	28.64 ± 6.85	19.49 ± 7.50	**<.001**
2CV PALS, %	27.04 ± 8.07	28.82 ± 8.13	25.17 ± 7.61	.**004**	27.39 ± 6.62	18.95 ± 6.83	**<.001**
Right heart							
FAC, %	43.77 ± 8.65	45.04 ± 8.71	42.50 ± 8.45	.**046**	43.21 ± 8.31	40.22 ± 8.70	.17
TAPSE, cm	2.14 ± 0.38	2.15 ± 0.37	2.13 ± 0.39	.76	2.17 ± 0.39	2.02 ± 0.39	.14
RVS, %	−20.30 ± −5.93	−20.59 ± −5.27	−19.98 ± −6.63	.56	−20.55 ± −6.24	−18.03 ± −7.76	.21

Abbreviations: ACE = Angiotensin‐converting enzyme; ARB = angiotensin 2 receptor; BMI = body mass index; 2CV = two‐chamber view; 4CV = four‐chamber view; COPD = chronic obstructive pulmonary disease; EDV = end‐diastolic volume; EF = ejection fraction; ESV = end‐systolic volume; FAC = fractional area change; PALS = peak of atrial longitudinal strain; PCI = percutaneous coronary intervention; LAD = left anterior descending; LAVoli = indexed left atrial volume; LCx = left circumflex; LM = left main; LV GLS = left ventricular global longitudinal strain; RCA = right coronary artery; RVS = right ventricular strain; TAPSE = tricuspid annular plane excursion.

The values in bold are statistically significant with *P* ≤ 0.05.

## 3 RESULTS

### 3.1 Population characteristics

**FIGURE 1:**
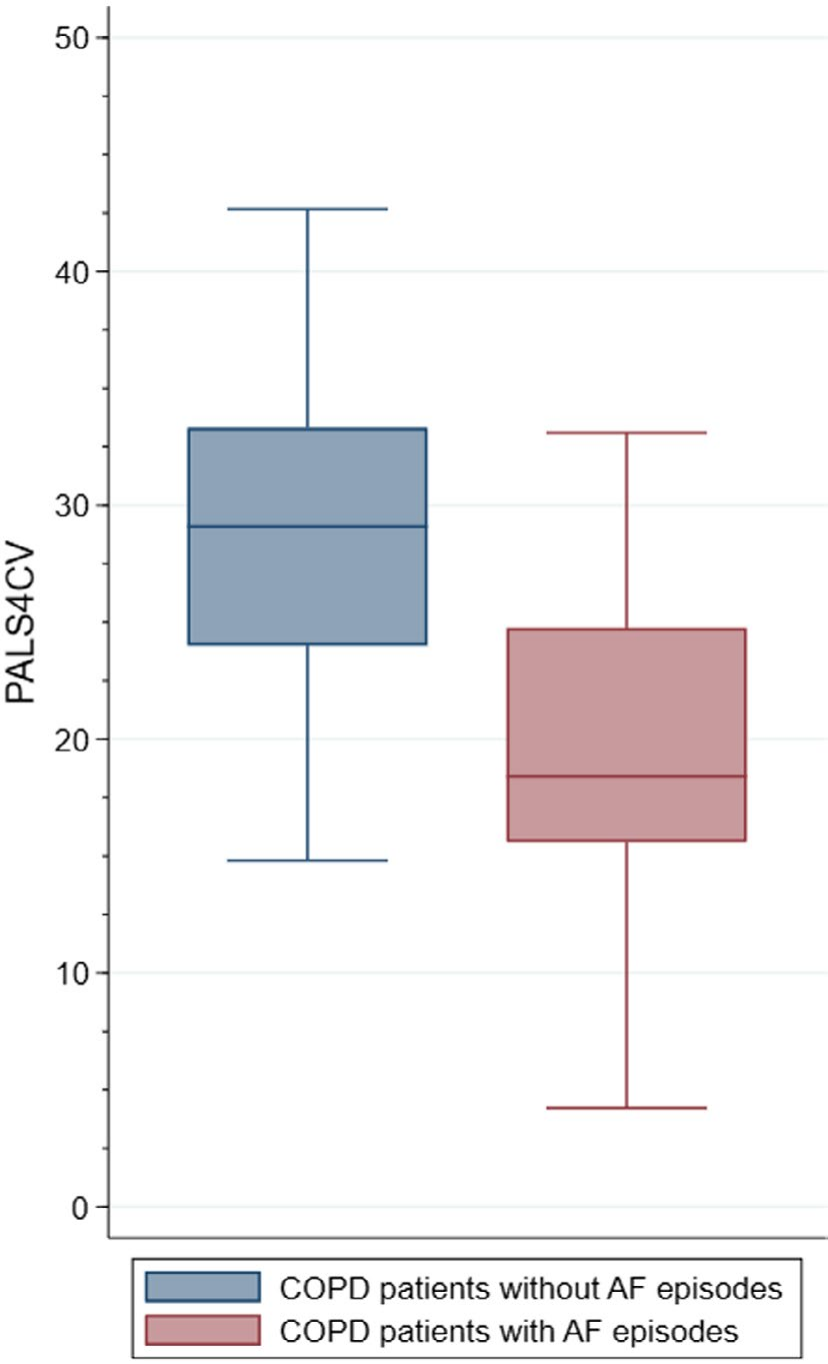
Box and whiskers plot displaying PALS values in COPD patients with or without atrial fibrillation in the follow‐up. Red box: COPD patients with AF episodes in follow‐up; Blue box: COPD patients without AF episodes in follow‐up. The images shows that PALS was significantly lower in patients with COPD who experienced AF. AF = atrial fibrillation; COPD = chronic obstructive pulmonary disease; PALS4CV = peak atrial longitudinal strain calculated in four‐chamber view

Overall, 175 patients have been included in the present analysis (Figure 1). From the original study population of 183 patients (137 patients of SCAP trial and 46 patients of NATHAN‐NEVER trial), we excluded eight patients because of several reasons: TTE was of sub‐optimal quality, pre‐existent diagnosis of AF, concomitant moderate or severe valve disease and presence of pacemaker or implantable cardiac device. Overall, 85% of patients were male with a mean age 67 ± 10 years. Ninety patients (51%) had confirmed COPD diagnosis (Table 1). Considering the cardiovascular risk factors, there was no statically significant difference among patients with and without COPD, except for a higher number of pack/years in COPD patients (*P* = .014, Table 1).

### 3.2 Peak atrial longitudinal strain and atrial fibrillation

**FIGURE 2:**
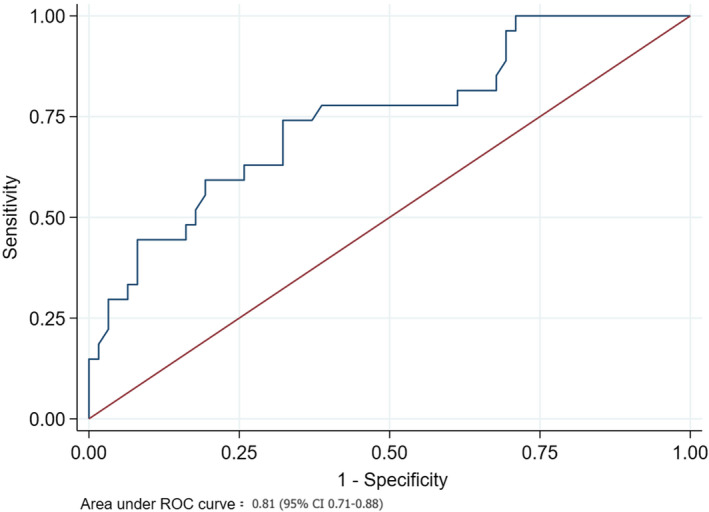
ROC curve analysis for PALS and atrial fibrillation. x‐axis = 1 ‐ specificity; y‐axis = sensitivity; ROC = receiver operating characteristic

**FIGURE 3:**
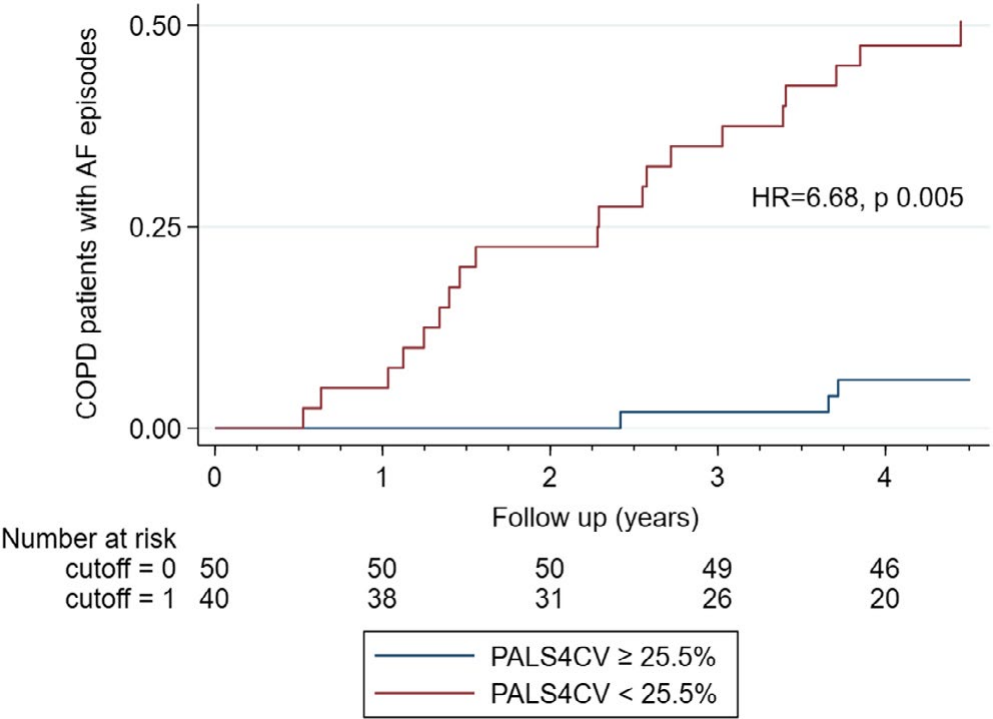
Atrial fibrillation occurrence according to the best PALS cutoff in COPD patients. Red lines: PALS <25.5%; Blu lines: PALS≥25.5%. AF = atrial fibrillation; COPD = chronic obstructive pulmonary disease; HR = hazard ratio; PALS = peak atrial longitudinal strain; PALS4CV = PALS calculated in four‐chamber view

PALS4CV was significantly lower in patients with COPD compared to patients without COPD (26% ± 8% vs. 30% ± 8% for PALS4CV, *P* = .003, Table 1). Considering the other echocardiographic parameters, LV global longitudinal strain (LVGLS), indexed left atrial volume, RVS, and FAC significantly differed in the two groups (Table 1). At the follow‐up, 30 patients experienced at least one episode of AF. Most of the events occurred in patients with concomitant COPD (26 events vs four events in patients with vs without COPD) (Table 1). Of note, in the same group occurred all the episodes of stroke (n = 7). Age at the time of the enrollment, body mass index (BMI), LV GLS, and PALS4CV (Figure 1) resulted as significant predictors of AF events at univariate Cox regression in COPD patients with CAD and were included in the multivariable model. At multivariable analysis, only PALS4CV (HR: 0.92, 95% CI: 0.86‐0.98, *P* = .014) resulted as an independent predictor of AF in COPD patients. The area under the ROC curve for PALS4CV and AF was 0.81 (95% CI: 0.71‐0.88), with the best cutoff value of 25.5% (sensitivity 87% and specificity 70%, Figure 2). Repeating multivariable Cox regression analysis with the PALS4CV cutoff <25.5%, this resulted as a strong predictor for AF (HR = 6.68, 95% CI 1.76‐25.31, *P* = .005, Figure 3).

## 4 DISCUSSION

The present study confirmed a high incidence of AF events in COPD patients with CAD. Also, PALS is altered and able to independently predict AF in a specific cohort of patients with acute (SCAP) or stable (NATHAN‐NEVER) CAD and COPD. Factors enhancing the risk of developing AF in COPD are related to hypoxia and hypercapnia, leading to vasoconstriction and pulmonary hypertension, resulting in right heart and diastolic dysfunction.^5^, ^13^, ^14^ At the same time, sympathetic overactivity and dynamic hyperinflation, obesity, and other concomitant cardiovascular risk factors such as hypertension facilitate the arrhythmogenic mechanism responsible for AF development.^13^ The additional value of the present analysis is the confirmation that PALS is a good predictor of AF development also in patients with COPD, independently by the presence of an acute or stable CAD, being the presence or not of acute coronary syndromes not significantly different among patients who develop AF in the follow‐up. The prevalence of AF in patients with COPD is estimated to be around 4% to 15%^15^ and increasing in patients with severe COPD. Considering the increased risk of stroke in patients with COPD^15^ where AF is one of the major determinants of stroke, this study points out the need to integrate a marker as PALS measurement in the echocardiographic workup of all COPD patients. Lower PALS values may help identify higher‐risk patients needing closer screening for AF to prevent ischemic complications. We may suppose that in this selected subgroup of patients loop recorder implantation or periodic electrocardiogram Holter monitoring could help identify early cases of AF and, subsequently, start therapy with novel oral anticoagulants. The limitations that the study presents are mainly related to the limited number of patients included. In the study population considered, the COPD degree was mainly mild or moderate,^9^, ^10^ thus justifying the limited number of AF events. However, the long follow‐up, the presence of a verified diagnosis of COPD with spirometry, and the application of comprehensive echocardiography to all patients are all points of strength that characterize this analysis. Finally, we did not analyze conduit and contractile phase of left atrial strain. PALS is a marker of atrial fibrosis as detected by cardiac magnetic resonance, and it is superior to other convectional parameters in predicting CV events.^8^ Thus, PALS seems to be an optimal surrogate marker of the atrial fibrosis that should be the reason why these patients are more prone to develop AF. Thus, we decided to use only this parameter even because it is the most standardized and reliable (Table 2).^16^, ^17^

**TABLE 2:** Univariate and multivariate analysis for atrial fibrillation in patients with CAD and COPD

	Univariate			Multivariate		
	HR	95% CI	*P* value	HR	95% CI	*P* value
Age at baseline	1.06	1.00–1.11	.**032**	1.05	0.99–1.11	.102
BMI	1.13	1.01–1.25	.**026**	1.13	0.99–1.29	.065
Active smoking habit	0.42	0.17–1.08	.071			
RAAS inhibitors	0.26	0.08–0.88	.**031**	0.19	0.02–1.57	.124
LVEF	0.97	0.93–1.00	.059			
LVGLS	0.88	0.81–0.95	.**001**	0.93	0.84–1.03	.142
PALS	0.88	0.83–0.93	**<.001**	0.92	0.86–0.98	.**014**

Note

Clinical and echocardiographic variables included in Table 1 with *P* < .1 after stratification for the presence of atrial fibrillation in patients with CAD and COPD were included in univariate analysis; only variables with *P* < .05 were included in multivariable model.

Abbreviations: BMI = body mass index; LVEF = left ventricle ejection fraction; LV GLS = left ventricle global longitudinal strain; PALS = peak atrial longitudinal strain; RAAS = renin angiotensin aldosterone system.

The values in bold are statistically significant with *P* ≤ 0.05.

## 5 CONCLUSIONS

Peak atrial longitudinal strain is altered and able to independently predict AF in a specific cohort of patients with CAD and COPD. This study points out the need to integrate a marker as PALS measurement in the echocardiographic workup of all COPD patients, to early identify those at high risk of AF development.

## CONFLICT OF INTERESTS

None declared.

## Data Availability

The data that support the findings of this study are available from the corresponding author upon reasonable request.
